# Comparison of the effect of light alcohol consumption on Japanese men with and without fatty liver

**DOI:** 10.3892/br.2020.1271

**Published:** 2020-01-13

**Authors:** Tasuku Hara, Yuya Seko, Naoto Iwai, Yutaka Inada, Toshifumi Tsuji, Takashi Okuda, Toshiyuki Komaki, Yoshito Itoh, Keizo Kagawa

Biomed Rep 11:191-198, 2019; DOI: 10.3892/br.2019.1242

Subsequently to the publication of the above article, the authors have noted that the Abstract and the Acknowledgements contained errors and an omission, respectively, and wish to correct these points for the record. First, in the Abstract, on p. 191, the pair of consecutive sentences beginning on line 16 should have read as follows (changes highlighted in bold and in the subsequent parentheses): “In the non-fatty liver group, the odds ratio (OR) for hypertension was 1.73 (1.04-2.88; P=0.035). In the fatty liver group, the OR for each Mets-related diseases were as follows: Dyslipidemia, 0.64 (0.44-0.95; P=0.028); impaired glucose tolerance, 0.57 (0.37-0.88; P=0.012); chronic kidney disease, 0.58 (0.36-0.94; P=0.029); and Mets by Japanese criteria, 0.63 (0.44-0.92; P=0.016). (“2”, featured erroneously after “1.04-2.88”, has been omitted). Secondly, in the Acknowledgements section, “Noriko” should have been properly acknowledged as follows: “The authors thank all the members of the Department of Gastroenterology and Hepatology, Fukuchiyama City Hospital. The authors would also like to thank **Ms. Noriko Nishikawa, of the medical division staff**, for assistance with data collection.”

The authors apologize for these errors, and for any inconvenience caused.

